# Current research into brain barriers and the delivery of therapeutics for neurological diseases: a report on CNS barrier congress London, UK, 2017

**DOI:** 10.1186/s12987-017-0079-9

**Published:** 2017-11-07

**Authors:** John Greenwood, Margareta Hammarlund-Udenaes, Hazel C. Jones, Alan W. Stitt, Roosmarijn E. Vandenbrouke, Ignacio A. Romero, Matthew Campbell, Gert Fricker, Birger Brodin, Heiko Manninga, Pieter J. Gaillard, Markus Schwaninger, Carl Webster, Krzysztof B. Wicher, Michel Khrestchatisky

**Affiliations:** 1 Institute of Ophthalmology, University College London, London, EC1V 9EL UK; 2 Department of Pharmaceutical Biosciences, Uppsala University, 751 24 Uppsala, Sweden; 3 Gagle Brook House, Chesterton, Bicester, OX26 1UF UK; 4 Centre for Experimental Medicine, Queen’s University Belfast, Belfast, Northern Ireland UK; 5 Department of Biomedical Molecular Biology, Ghent University, Ghent, Belgium; 6 VIB-UGent Center for Inflammation Research, VIB, Ghent, Belgium; 7 School of Life, Health and Chemical Sciences, Open University, Milton Keynes, UK; 8 Smurfit Institute of Genetics, Lincoln Place Gate, Trinity College Dublin, Dublin 2, Ireland; 9 Institute of Pharmacy and Molecular Biotechnology, Ruprecht-Karls University, Heidelberg, Germany; 10 Department of Pharmacy, Faculty of Health and Medical Sciences, University of Copenhagen, Copenhagen, Denmark; 11 NEUWAY Pharma GmbH, Ludwig-Erhard-Allee 2, 53175 Bonn, Germany; 12 2-BBB Medicines BV, Leiden, Netherlands; 13 Institute of Experimental and Clinical Pharmacology and Toxicology, University of Lübeck, Lübeck, Germany; 14 Antibody Discovery and Protein Engineering, MedImmune, Cambridge, UK; 15 Ossianix Inc., Stevenage, UK; 16 CNRS, NICN, Aix Marseille Univ, Marseille, France; 17 Vect-Horus, Faculte de Medecine Nord, 51 Boulevard Pierre Dramard, Marseille, France

**Keywords:** Blood–brain barrier, Blood–CSF barrier, Blood–retinal barrier, Neuroinflammation, Viral vectors, Drug delivery, Antibody therapy, MicroRNA, Liposomal technology, Protein capsules

## Abstract

This is a report on the CNS barrier congress held in London, UK, March 22–23rd 2017 and sponsored by Kisaco Research Ltd. The two 1-day sessions were chaired by John Greenwood and Margareta Hammarlund-Udenaes, respectively, and each session ended with a discussion led by the chair. Speakers consisted of invited academic researchers studying the brain barriers in relation to neurological diseases and industry researchers studying new methods to deliver therapeutics to treat neurological diseases. We include here brief reports from the speakers.

## Background

The blood–brain barrier (BBB), and the other blood–tissue barrier sites of the central nervous system (CNS), have been the subject of extensive research since their discovery over 100 years ago. Despite considerable advances in our understanding of the structural and functional interface of the BBB, there remain many gaps in our knowledge particularly regarding its role in disease and the challenges it presents to therapeutic intervention. In recent decades the classic concept of the BBB has also evolved such that it now cannot be considered in isolation from other cellular components of the CNS. Accordingly, the emergence of the concept of the neurovascular unit (NVU) has re-shaped our approach to studying the BBB. In addition, other blood–tissue interfaces, such as the blood–cerebrospinal fluid and blood–retinal barriers, are also providing additional insight into the communication between the blood and the CNS.

Our understanding of the normal structure and function of the blood–CNS barriers is well advanced but their roles in many diseases remains incomplete. Whereas blood–CNS dysfunction in some conditions is evident, such as in tumours, multiple sclerosis and stroke, in other diseases such as Alzheimer’s disease, Parkinson’s disease and epilepsy the involvement is less obvious. Indeed, gross changes, such as loss of structural integrity have clear pathological consequences, whereas subtle changes to function may be more difficult to ascertain and place within the overall pathogenesis of a disease. Whether cause or effect, therapeutic targeting of barrier dysfunction remains an attractive proposition and drives much of the translational research currently underway. However, various questions concerning barrier susceptibility to disease remain outstanding. These include the heterogeneity of the vasculature within the CNS and as a consequence its differential response. Indeed, it is known that in the normal BBB there is endothelial cell heterogeneity that is not only dependent on its position throughout the vascular bed (i.e. artery versus arteriole, versus capillary, versus venule, versus vein) but also within the same region of the vasculature. Moreover, the barrier within different structures of the CNS also differs and together such heterogeneity will undoubtedly impact on the variable response of the barrier to disease. For example, the microvascular pathology observed in diabetes is far more pronounced in the retina than in the brain, the response of white matter vessels and those in grey matter differ in multiple sclerosis, and in meningitis it is the meningeal vessels that are susceptible.

Aside from the direct relationship between barrier dysfunction and disease pathogenesis there is another longstanding and major challenge facing those working in the field. This relates to the problems posed by a structurally intact barrier that restricts the delivery of therapeutics to the brain. For almost 50 years this has proved to be largely insurmountable and only recently have advances been made that provide some optimism.

In this CNS barrier congress experts from various disciplines were brought together to collectively discuss the best ways to overcome these challenges, and pave the way for progress in the treatment of neurological disease. In recent years, our understanding of barriers has undergone re-evaluation and during the meeting various pressing questions were discussed. These included the role that non-endothelial cells in the NVU play in blood–brain barrier regulation, how much barrier dysfunction really occurs in different CNS diseases, why regional differences exist, and how do immune cells impact barrier function. Contrary to previous dogma, the CNS barriers are now recognised as complex, dynamic, interactive structures that contribute to disease on many levels. Recent advances in drug delivery technologies to the CNS were also presented and discussed at length. Pioneering groups have been perfecting new methods to ferry drugs across the CNS barriers, particularly the blood–brain barrier where access via other routes is problematic. Accordingly, the latest developments in liposome, peptide, antibody, and nanoparticle technology for therapeutic delivery were showcased and how far these technologies have to go before they can become widely available was discussed.

This CNS barrier congress allowed for the presentation of unpublished data, the exploration of new technologies, and provided a select platform for academic and industrial leaders in the field to form collaborations, exchange ideas and identify new strategies for development.

## The specialised vascular barriers of the CNS and their influence on leukocyte migration

### John Greenwood

The specialised vascular endothelial cells that line the vessels of the brain and retina form an impermeable but selective barrier between the blood and the neural parenchyma. Under normal physiological conditions this critical interface, termed the blood–brain/retinal barrier, strictly limits the passage of solutes and cells between these two compartments. During disease, however, the endothelial cells become activated resulting in a change of phenotype and an alteration in their regulatory function. Thus, in neuroinflammatory diseases such as multiple sclerosis and posterior uveitis, the function of these vascular barriers changes resulting in an enhanced influx of leukocytes. Accordingly, the endothelial cells of the CNS are recognised as playing a pro-active role in the propagation, maintenance and possibly resolution of CNS inflammatory lesions. Over the last few decades increasing evidence, from our laboratory and that of our collaborators, has shown that the endothelial cell responds to adherent leukocytes in variety of ways resulting in immediate facilitation of diapedesis to the longer-term regulation of gene expression. Many of these outside-in signaling cascades are generated through the engagement of endothelial immunoglobulin superfamily adhesion molecules such as ICAM-1, which act as signal transducers leading to the activation of the small GTPase rho, eNOS, phospholipase C, protein kinase C, src kinase and release of intracellular calcium [1]. In addition, we have reported more recently that downstream activation of MAP kinases, re-arrangements of the actin cytoskeleton and tyrosine phosphorylation of various cytoskeletal associated proteins results in the activation of divergent inflammatory outcomes [2]. Finally, we have established that the tightness of the endothelial cell junction and cell cytoskeletal stiffness dictates the route of leukocyte transmigration [3]. Deciphering the end-points of these signaling networks and identifying potential pharmacological targets, remains a major focus of the laboratory.

## Non-VEGF mediated breakdown of the blood–retinal barrier: alternative strategies to treat diabetic macular oedema

### Alan W. Stitt

Diabetic macular oedema (DMO) occurs as a symptom of diabetic retinopathy and often leads to significant vision-loss [4]. The condition is characterised by progressive breakdown of the blood retinal barrier (BRB) as a result of tissue ischaemia and/or inflammation which drive imbalances in vasoactive cytokines and growth factors causing compromise of normal neuroglial–vascular interactions and endothelial dysfunction [4]. Neutralisation of vascular endothelial growth factor (VEGF) using intravitreal injection of humanised antibodies has become a mainstream treatment for DMO. Unfortunately this is not effective for all DMO patients [5] and there is a need for additional therapeutic approaches which could be used instead of, or in conjunction with, current anti-VEGF drugs.

Our groups have recently focused on the permeability-inducing agent lysophosphatidylcholine (LPC) which is produced through activity of the enzyme lipoprotein-associated phospholipase A_2_ (Lp-PLA_2_). Using a range of in vitro and in vivo approaches, we have recently shown that inhibition of Lp-PLA_2_ can prevent diabetes-induced compromise of the BRB in a manner that is comparable to intravitreal VEGF neutralisation [6]. Importantly, these protective effects were additive when both targets were inhibited simultaneously. Our mechanistic studies also demonstrated that LPC potently induced permeability, and that there was a coalescence of the LPC and VEGF pathways via a common VEGF-receptor-2 mediated mechanism [6, 7]. We have concluded that Lp-PLA_2_ may be a useful therapeutic target for patients with DMO, perhaps in combination with currently administered anti-VEGF agents. Such studies demonstrate the utility of studying “real-world” clinical scenarios whereby new approaches can be evaluated alongside the current gold-standard therapies and offer hope for patients who are non-responsive to current treatment regimes.

## The choroid plexus is an important player in the induction of neuroinflammation

### Roosmarijn Vandenbrouke

The choroid plexus epithelium which forms the blood–CSF barrier is a unique single layer of epithelial cells situated at the interface between blood and cerebrospinal fluid (CSF). The choroid plexus epithelium has several different important functions: it forms a barrier to protect the brain from fluctuations in the blood, produces CSF and is responsible for the active removal of toxic molecules from the brain and thereby assures brain homeostasis. In recent years, the choroid plexus epithelium has gained increasing attention, especially its role in different pathologies. Indeed, subtle changes in the choroid plexus epithelial cells will result in changes in CSF composition, exerting wide-ranging effects on the brain and subsequently affecting disease progression. Therefore, understanding blood–CSF barrier functionality under physiological and pathophysiological conditions might open up new therapeutic strategies to treat inflammatory brain diseases.

Our research focusses on the effect of both systemic inflammation (sepsis) and neuroinflammation (the age-related disease Alzheimer’s disease) on the blood–CSF barrier. More specifically, we study key molecules that play a role in the activation of detrimental processes at the blood–CSF barrier upon inflammation, focussing on barrier integrity and secretory activity. These studies might allow us to identify novel therapeutic strategies to prevent neuroinflammation.

To address the effect of sepsis, we used intraperitoneal lipopolysaccharide (LPS) injection and the caecal ligation and puncture (CLP) mouse model, while Alzheimer’s disease was studied using the intracerebroventricular (icv) injection of soluble amyloid β oligomers (AβO) in mice. Our studies showed that both systemic [7] and central inflammation [8] induce increased blood–CSF barrier leakage. Interestingly, these effects could be attributed to matrix metalloproteinase (MMP) activity. Additionally, peripheral inflammatory triggers induced an increase in extracellular vesicle (EV) release by the choroid plexus epithelium into the CSF [9]. Detailed analysis by electron microscopy and inhibitor studies using the neutral sphingomyelinase 2 inhibitor GW4869, revealed that especially the biogenesis of exosomes is increased upon systemic inflammation. Strikingly, these choroid plexus-derived EVs are able to enter the brain parenchyma, are taken up by astrocytes and microglia and transfer a pro-inflammatory message to the brain [9]. Interestingly, we observed that the icv injection of AβO has similar effects on the extracellular vesicle production of the choroid plexus epithelial cells.

Clearly, our data show that both peripheral and central inflammatory triggers affect barrier and secretory activity of the choroid plexus epithelium and these results might open up new therapeutic strategies to treat neuroinflammatory diseases.

## MicroRNAs and blood–brain barrier function in multiple sclerosis

### Ignacio A. Romero

Blood–brain barrier dysfunction is a major hallmark of many CNS disorders such as multiple sclerosis. BBB breakdown is characterised by three main features: (1) increased permeability across the endothelium; (2) alteration in the expression of cell-surface receptors and/or transporters; and (3) activation of endothelial cells to support leukocyte extravasation into the CNS parenchyma. Many of these cerebrovascular pathophysiological effects are underpinned by overt acute or chronic changes in gene expression in cerebral endothelial cells and in other cells of the NVU.

MicroRNAs (miRs) are novel regulators of gene expression at the post-transcriptional level and may potentially play a key role in cerebrovascular pathophysiology. MiRs mainly suppress the expression of target genes either by blocking translation or by inducing mRNA degradation. We have first identified miRs whose levels in endothelial cells change following inflammatory stimuli. Inflammation induces upregulation of several key inflammatory miRs termed inflammiRs (miR-155 and miR-146) in brain endothelial cells. By contrast, there are other miRs termed brain endothelial housekeeping miRs (miR-125b and miR126) whose levels are elevated under quiescent conditions but are significantly reduced by pro-inflammatory cytokines [10–12]. These inflammation-induced changes in the finely-tuned balance of cerebral endothelial miR levels promote cerebrovascular dysfunction. For example, miR-155 contributes to BBB leakiness by reducing expression of tight junctional and focal adhesion components but it also promotes leukocyte firm adhesion to brain endothelium by indirectly increasing expression of cell adhesion molecules. Conversely, miR-146 inhibits leukocyte firm adhesion to brain endothelium by suppressing expression of key activators of the NF-κB pathway. Brain endothelial miRs could potentially be considered for targeted prophylaxis and therapies for BBB dysfunction.

However, we are still far from validating cerebrovascular miRs as potential therapeutic or prophylactic targets for neurovascular dysfunction in inflammatory and/or autoimmune disorders. First, there is the likelihood that several different miRs have combinatorial effects in specific CNS pathologies. Second, individual miRs likely have multiple gene targets and effects on other endothelial cellular pathways that contribute to the pathogenesis of inflammatory disease. In addition, non-specific delivery of miR modulators into other tissues or organs (e.g. liver, kidney) could cause unwanted side effects unless very specific delivery systems targeted at the cerebrovascular bed are available, Nevertheless, the potential for manipulating this novel class of regulators of gene expression for therapeutic purposes is huge and should be given considerable attention in the near future.

## Dose dependent expression of claudin-5 is a modifying factor in neurological diseases

### Matthew Campbell

Abnormalities in neuronal functioning in psychiatric conditions may derive in part from abnormalities in blood vessel–neuron interactions [13]. We have shown that the claudin-5 gene, a central component of the paracellular pathway of the BBB is associated with schizophrenia in individuals with the chromosomal disorder 22q11 deletion syndrome (22q11DS), a condition that confers a 30-fold increased lifetime risk of developing schizophrenia. A variant in this gene results in up to 50% less protein product and a targeted suppression of claudin-5 in distinct brain regions, or in an inducible “knockdown” mouse, identifies strong phenotypic correlations with schizophrenia. Additionally, post-mortem human schizophrenia donor brain tissues show evidence of BBB dysfunction, while some of the most common anti-psychotic drugs can directly regulate claudin-5 expression. Identifying the underlying cause of neuropsychiatric conditions at the level of the BBB suggests novel drug entities targeting the integrity of the BBB may have utility in treating these debilitating and socially isolating condition [14, 15]. All work involving animals was approved by the institutional ethics committee and all national licences were in place prior to commencement of work.

## Impact of ABC transporters on blood–brain barrier function

### Gert Fricker

The CNS requires a well-balanced homeostasis. Therefore, it is protected by the BBB, which is set up by endothelial cells of brain microvessels. These cells act as a dynamic regulator of ion balance, mediator of nutrient transport and barrier to harmful molecules. A central role accords to ATP-binding-cassette (ABC) export proteins, predominantly P-glycoprotein (P-gp, ABCB1), breast cancer resistance protein (BCRP, ABCG2) and multidrug resistance related proteins (MRPs, ABCCs). ABC transporters are predominantly expressed in microvessel endothelial cells, but are also located on other cell types, such as astrocytes, microglia, and neurons. In microvessel endothelial cells ABC transporters exhibit a polar distribution. P-gp is primarily found in luminal membranes, however, there is evidence that it also localizes to a certain amount to abluminal membranes as well as to pericytes and astrocytes. Studies using a fluorescent labeled construct of P-gp indicate that the export pump is not organized as a single molecule within membranes but forms clusters of several proteins close together [16]. BCRP appears to be predominantly located at the luminal surface of endothelial cells. Mrps are also expressed at the BBB, however, there is still considerable discussion about the extent of expression, involvement in drug transport and subcellular localization. Inhibition of ABC transporters significantly alters the brain disposition of transporter substrates as illustrated by studies in human: positron emission tomography (PET)-imaging revealed that cyclosporin A modulation of P-gp increased transfer of verapamil into the brain. In another PET-trial uptake of loperamide into the CNS was enhanced after inhibition of efflux pumps by tariquidar.

Various mechanisms control expression and function of ABC transporters. Regulation occurs either by ligand-activated transcription factors or by external stress signals which modulate ABC transporter function. The nuclear receptors PXR (pregnane X receptor), AhR (arylhydroxycarbon-receptor), CAR (constitutive andro-stane receptor), VDR (vitamin D receptor) and PPAR-γ (peroxisome proliferator-activated receptor) and related signaling events are of particular interest, since they bind many xenobiotics and subsequently upregulate ABC-transporter expression. ABC transporters also contribute to various CNS diseases. For example, brain tissue from Alzheimer patients showed an inverse relationship between P-gp expression and disease progress and inhibition of P-gp in a rodent Alzheimer model increased amyloid-β levels in brain. Studies showed that St. John’s Wort administration to mice resulted in significant reductions of parenchymal amyloid-β accumulation as well as in a moderate increase in cerebrovascular P-gp expression [17]. In summary, ABC-transporters are of outstanding relevance for the proper function of the blood–brain barrier. They are very sensitive to exogenous and endogenous stimuli and are involved in the progression of various CNS diseases.

## In vitro models of the blood–brain barrier, pro’s and con’s

### Birger Brodin

The brain capillary endothelium serves as a gateway for exchange of nutrients, hormones and metabolites between plasma and brain parenchyma, and acts as a barrier for CNS uptake of the majority of drug compounds. Mechanistic in vivo investigations of drug and nutrient transport, signalling and metabolism in the brain endothelium can be difficult to perform, since the brain endothelium is embedded within a complex structure*, the neurovascular unit*, itself within the multi-compartment brain and fluid system.

Cell isolation and culture protocols for growing brain endothelial cells in monolayers, either in monoculture or in co-culture with other cell types of the NVU, have been developed over the last 40 years. Although no ideal cell culture model of the blood–brain barrier is yet available, the in vitro models have evolved to become useful tools in the studies of barrier biology and drug delivery.

In vitro models based on primary cell cultures of animal origin (typically bovine, porcine or murine models) display endothelial cell morphology, expression of BBB tight junction proteins and high transendothelial electrical resistance, subject to astrocyte induction [18]. They display vectorial transport of efflux transporter substrates, indicative of luminal expression of ABC-type pumps [19]. The major drawback of these models may be downregulation of a number of solute carrier family proteins (SLC’s) as compared to the expression in vivo. Alternatively, immortalized cell lines of mouse, rat and human origin, in general without astrocyte induction, tend to be considerably more leaky than the in vitro models based on primary cell cultures. Some immortalized cell lines do, however, show relatively higher expression of some BBB-specific SLC transporters than the models based on primary cell cultures and may serve as excellent tools for studies of uptake, receptor activation and some signal transduction systems. A recent approach, generation of in vitro models of the BBB using human stem cells, has resulted in cell monolayers with both high monolayer tightness and expression of BBB-specific marker proteins [20]. However, the stem cell cultures are not yet widely used, and have not been fully validated with respect to transport properties and functional transporter profiling.

In brief, established in vitro blood–brain barrier models all have their advantages and drawbacks. Primary cell lines of bovine, porcine and rodent origin generate tight monolayers whereas immortalized cell lines may have higher transporter expression levels but less tightness. Models derived from human stem cells shows great promise, but are not yet fully characterized.

## Measuring blood–brain barrier transport of drugs—the hurdle in drug discovery and development

### Margareta Hammarlund-Udenaes

Measurement of total drug levels in the brain has been a common but unhelpful practice for many years in drug discovery programs aiming at central drug effects. The paradigm has changed with the introduction of the pharmacologically more important unbound brain interstitial fluid to unbound blood concentration ratio, K_p,uu,brain_, and with more high-throughput methods to estimate this parameter (the brain slice and the brain homogenate techniques).

By combining several of these measurements, the combinatory mapping approach (CMA) allows estimation of not only BBB transport, but also intracellular distribution of the pharmacologically active, unbound drug moiety [21]. CMA can also be used to assess possible lysosomal accumulation that may predict phospholipidosis as a serious side effect. The technique allows estimation of brain regional differences in BBB transport and binding, something that can influence effect/side effect patterns of drugs. In a study of six antipsychotic drugs, we found very different BBB and intracellular distribution patterns of the drugs [22]. There was a sixfold difference in regional BBB transport of risperidone (a P-glycoprotein substrate), with a more efficient efflux in cerebellum than in frontal cortex in rats, while other drugs such as quetiapine and clozapine showed very small differences in regional transport. In a separate study utilizing the CMA, we did not find any change in the BBB transport of five selected drugs in disease models of Alzheimer’s disease (ArcSwe) and Parkinson’s disease (A30P) compared with wild-type mice, counteracting the view that the BBB is leaky in disease.

Microdialysis was used to study if liposomal delivery would improve the brain uptake of methotrexate [23]. With microdialysis it was possible to separate the liposomally-encapsulated drug from released drug in blood, and to measure the released compound in brain. While liposomes based on hydrogenated soy phosphatidylcholine did not change BBB uptake at all compared with administering the free drug, the egg-yolk phosphatidylcholine liposomes increased the uptake into the brain threefold, reaching concentrations that could be pharmacologically active also in humans.

In conclusion, there are three main conceptual parts of brain drug transport, the rate of transport, measured as the permeability surface area product, the extent of transport measured with K_p,uu,brain_, and the nonspecific intra-brain binding, all contributing to different aspects of brain drug delivery. The extent of transport is considered clinically most relevant, as it measures the steady state relationship across the BBB, quantifying active efflux and influx processes.

## NEUWAY pharma: qualified for CNS delivery

### Heiko Manninga

NEUWAY Pharma GmbH, a German based biotech company, is focusing on the preclinical and clinical development of innovative therapeutics for treatment of brain diseases based on its proprietary CNS Drug Delivery Platform.

**Fig. 1:**
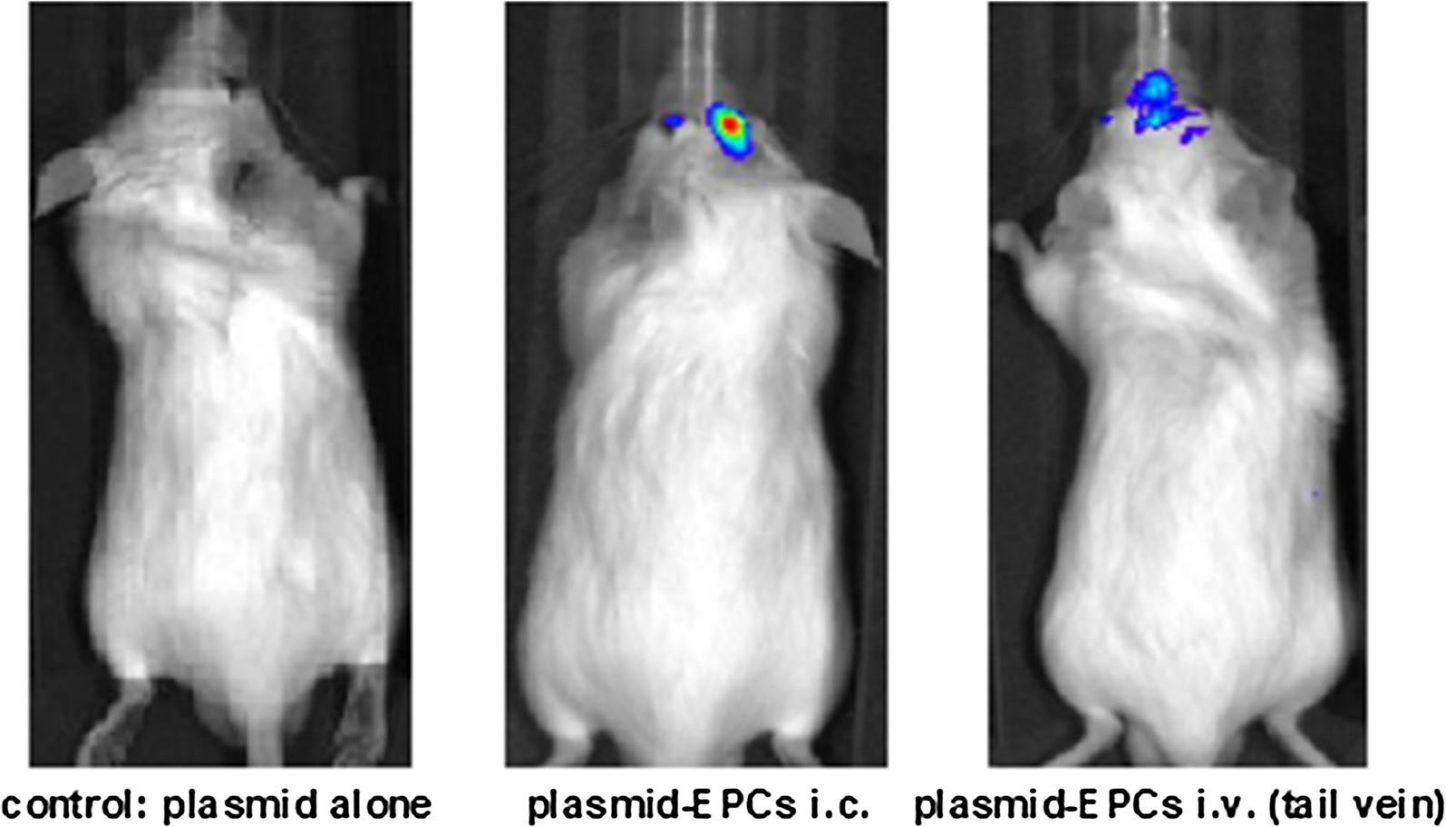
The enzyme luciferase produces light when the conversion of luciferin occurs. If a plasmid coding for luciferase is administered intravenously, no light signal can be detected (left mouse). The enzyme was degraded in the bloodstream. If the luciferase plasmid packaged in Engineered Protein Capsules (EPCs) is applied directly into the brain (intracerebral, i.c., middle mouse), a luminous signal can be detected there after administration of luciferin. This is also observed when the luciferase plasmid is packaged in EPCs and injected intravenously (i.v., right mouse)

The presented platform is based on a protein derived from the JC-Virus, which naturally forms capsules, named Engineered Protein Capsules (EPCs). EPCs may be used as carrier to transport highly active drug substances—ranging from small molecules to large nucleic acid strands—across the intact BBB. NEUWAY has demonstrated that its EPC-based proprietary CNS drug delivery platform can deliver plasmids over the blood brain barrier into CNS cells leading to gene expression in the brain. In a proof of concept study it was shown that intravenous injection of EPCs loaded with DNA encoding for luciferase induced an activity of the resulting enzyme, which was detected by bioluminescent signals in the brain (Fig. 1). More accurate studies of the brains of such treated mice clearly showed that the signals from the brain cells are beyond the blood–brain barrier and not from other cells, e.g., blood vessels.

As EPCs can carry large nucleic acid strands, they may be useful for gene therapy of rare diseases. NEUWAYs current development focuses on lysosomal storage diseases, like metachromatic leucodystrophy. For this and other rare diseases, NEUWAY plans to run its own clinical development programs. NEUWAY is also open to partner its CNS drug delivery platform with pharmaceutical or biotech companies preferably if large indications, like Alzheimer’s disease, are addressed.

Investigations conformed to the Guide for the Care and Use of Laboratory Animals published by the US National Institutes of Health (NIH Publication No. 85-23, revised 1996).

All animal procedures were approved by the appropriate state agency (Protocol Number W17/11).

## Liposomal technology and the blood–brain barrier

### Pieter J. Gaillard

2-BBB is successfully harnessing liposomal technology to mediate safe targeting and enhanced drug delivery to the brain. The G-Technology^®^ platform has been shown to enhance transport of therapeutics across the blood–brain barrier. Preclinical studies with the lead product, 2B3-101, containing doxorubicin, demonstrated significantly enhanced delivery and improved survival of mice in comparison to currently available compounds.

Phase I clinical trials with 2B3-101 have been completed, with Phase IIa set to determine preliminary antitumor efficacy at the maximum tolerated dose. The study population of this phase I/IIa trial consisted of patients who met all of the inclusion criteria and none of the exclusion criteria, and provided written informed consent. The protocol, any amendments and all other applicable study documents for the study were reviewed by the Independent Ethics Committees (IECs) of the following countries: NL: The “Nederlands Kanker Instituut Antoni van Leeuwenhoek Ziekenhuis”; BE: The “Association Hospitaliere de Bruxelles-Centre des Tumeurs de l’ULB”; USA: The “Office of Human Research Ethics of the University of North Carolina at Chapel Hill”; FR: The “Comite De Protection Des Personnes Ile De France III”. Clinical trial identifier is NCT01386580.

2-BBB’s second product in development, 2B3-201, containing methylprednisolone, is designed to treat patients with acute neuroinflammation. Superior efficacy, reduced side effects and enhanced plasma circulation half-life have been shown in rodent models, in comparison to competitive compounds. A phase I study in healthy volunteers was completed, addressing safety, tolerability and pharmacokinetics, and markers for pharmacological proof-of-concept. Collectively, preclinical and clinical evidence to date has demonstrated that G-Technology^®^ offers a promising platform to safely enhance delivery of drugs to the brain. The study was approved by the Medical Ethics Committee of the BEBO Foundation (Assen, The Netherlands). Subjects provided written informed consent. Clinical trial identifier is NCT02048358.

## The blood–brain barrier in gene therapy: hurdle or target?

### Markus Schwaninger

**Fig. 2:**
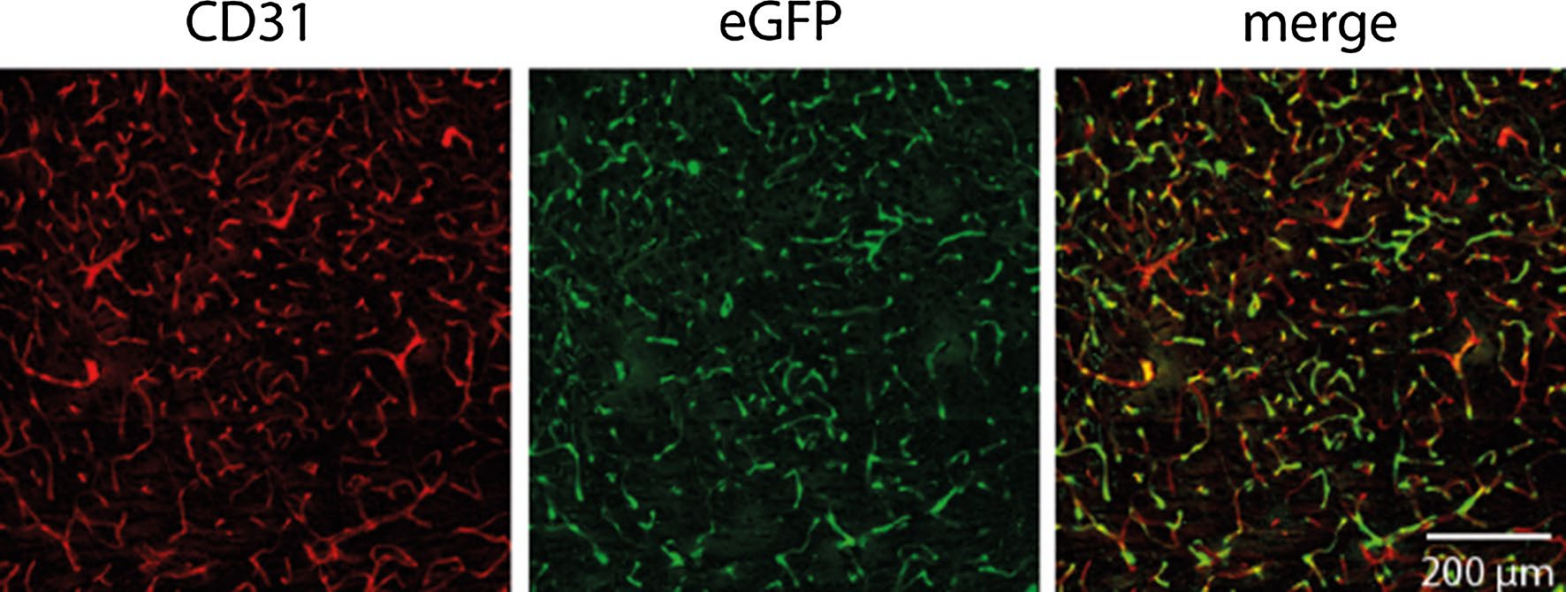
After intravenous administration of the vector AAV-BR1-eGFP (enhanced Green Fluorescent Protein) to mice most brain endothelial cells expressed eGFP. eGFP expression in other tissues was low. In exchange of eGFP other genes can be selectively expressed in brain endothelial cells. The figure shows a representative section of the thalamus stained for the endothelial cell marker CD31 and eGFP

Gene therapy provides attractive therapeutic options for the many diseases for which no treatment is still available. The adeno-associated virus (AAV) is a safe and efficient tool to transfer genes. However, conventional AAV-based vectors do not cross the BBB when administered systemically. This obstacle can be solved by administering vectors either intrathecally or using vectors based on AAV serotype 9 that cross the BBB to a limited extent. Still another approach is to target the BBB itself. In order to develop a brain endothelial selective vector, a novel strategy for in vivo screening of random ligand libraries displayed on viral capsids was used [24]. Several rounds of in vivo selection resulted in AAV-BR1, an AAV vector with unprecedented selectivity for brain endothelial cells after systemic intravenous administration. Due to its specific features, this vector allows for modulating and repairing the BBB. Its efficacy was tested in a mouse model of the hereditary disease incontinentia pigmenti [25], in which a deficiency of the Nemo gene leads to a loss of brain endothelial cells and breakdown of the BBB. Consequently, patients suffer from neurological disability and epileptic seizures. Intravenous injection of AAV-BR1 transferring the Nemo gene was able to largely reduce endothelial cell death and to ameliorate disruption of the BBB. Mice treated with AAV-BR1-Nemo showed less activation of astrocytes. Importantly, the occurrence of focal epileptic seizures was significantly reduced by the gene therapy [26]. Probably due to its high brain endothelial selectivity, the vector did not induce hepatocellular carcinoma or other adverse effects that have been observed in rodents during gene therapy with AAV vectors. Previous studies suggest that transduced brain endothelial cells may release enzymes that are missing in the CNS [27]. Thus, transducing brain endothelial cells with gene vectors offers the opportunity to overcome the BBB and to supply diverse proteins to the diseased brain (Fig. 2). The conceptual progress of this approach is that the BBB is no longer considered as a hurdle but as the target of a successful therapy.

## Antibody therapeutics for CNS diseases and their delivery across the BBB

### Carl Webster

The BBB protects and regulates the homeostasis of the brain. However, this barrier also limits the access of drugs, including large molecule therapeutics, to the brain and results in sub-therapeutic concentrations of drug reaching CNS targets. MedImmune have utilised their antibody engineering platform to develop a fully human monoclonal antibody that targets the blood brain barrier to deliver therapeutics to the CNS. The antibody was isolated from a phage display library by competitive elution using the known BBB transporting antibody FC5. A combination of pharmacodynamic (PD) and pharmacokinetic (PK) assays were used to confirm central penetration. In mice, the peripheral PK was in line with the expected half-life for a human antibody and showed no target mediated clearance at doses from 0.45 to 45 mg/kg. Brain penetration was around 3% of injected dose and persisted beyond 7 days. To confirm the antibody present in the CNS was able to access central targets, a fusion to interleukin-1 receptor antagonist (IL-1RA) was made. IL-1 is mediator of neuropathic pain and its antagonism is analgesic. Partial ligation of the sciatic nerve results in a neuropathic pain phenotype that manifests through increased sensitivity to mechanical pressure on the hind paw. Peripheral administration of the BBB-IL-1RA fusion resulted in dose dependent analgesia, confirming the ability of the BBB antibody to penetrate the CNS. However, the doses required were high and this fusion protein would not represent a commercially viable treatment for neuropathic pain. Other molecular targets represent better points of intervention in the pain signalling pathways and therefore an antibody against the ATP gated ion channel P2X4 was developed. P2X4 regulates the physiology and pathophysiology of spinal microglia and is implicated in pain signalling. Channel blocking antibodies against mouse P2X4 were obtained by immunising rats with recombinant mouse P2X4. Approximately 5000 hybridoma lines binding to P2X4 were identified of which 28 showed blocking activity. Lead antibodies were tested by intrathecal administration to mice where analgesia in the neuropathic pain model was demonstrated. Peripheral administration resulted in analgesia only when fused to the BBB antibody, confirming central exposure of the drug molecule was required. Doses as low as 3 mg/kg produce statistically significant analgesia, and this offers hope of a treatment to many people suffering from neuropathic pain.

All procedures described here were performed in accordance with the Animals (Scientific Procedures) Act 1986 and were approved by the MedImmune local ethics committee.

## Harnessing bispecific antibodies to overcome the blood–brain barrier

### Krzysztof B. Wicher

Penetration of the BBB remains a significant impediment in development of biologics for CNS-related diseases. To identify efficient brain-shuttles, we used shark single domain antibody phage libraries in a combined in vitro and in vivo selection process. To achieve the highest discovery rate of brain-penetrant clones, we employed next generation sequencing of phagemid DNA after subsequent rounds of selection. This way, we identified a panel of TfR1-specific shark antibodies, which are efficiently transported to mouse brain parenchyma. One of these antibodies (B2) fused to human IgG1 Fc showed more than 12-fold better brain uptake than the control, reaching therapeutic concentrations (5 nM) upon single intravenous injection of 25 nmol/kg (~ 1.9 mg/kg). Most of the brain-associated B2-hFc was found in brain parenchyma. Immunohistochemistry analyses showed the protein present both in the intestinal fluid and in the cells, including neurons. B2 antibody is safe upon administration of high doses in mice, as manifested by lack of significant acute adverse reactions or changes in blood morphology of injected animals. It binds to human TfR1 with comparable affinity as to mouse TfR1, but does not bind to TfR2. Moreover, in silico analysis indicates that B2 antibody would have relatively low immunogenic properties in humans.

We next constructed several different variants of bi-specific antibodies composed of B2 and rituximab, a well characterized anti human CD20 specific antibody used to treat peripheral B cell lymphomas. Many of these variants shuttle efficiently to brain and provide up to 14× better exposure than original antibody. The hybrid proteins retain their binding to both TfR1 and CD20 and at least some of them appear to mediate antibody-dependent cell cytotoxicity response on human CD20^+ve^, but not on CD20^−ve^, cells, similar to that of rituximab. Thus, they offer possible therapeutics for multiple sclerosis and cerebral B-cell lymphoma.

All research procedures/experiments described here were performed in accordance with Animals Scientific Procedures Act 1986 and European Directive 2010/63/EU. All studies performed were approved by the Royal Veterinary College Animal Welfare and Ethics Review Body and comply with the UK Home Office guidelines and codes of conduct.

## Drug delivery across the blood–brain barrier using peptide conjugates

### Michel Khrestchatisky

Drug delivery to the brain is hindered by the BBB. Receptor-mediated transport/transcytosis (RMT) can be used to shuttle therapeutics into the brain in a non-invasive manner. We developed peptide- and nanobody-based ligands that target specific receptors and that can be used as vector molecules to transport drugs or imaging agents across the BBB. Members of the low density lipoprotein receptor (LDLR) family appear relevant to deliver drugs into cells and organs and we report results on the development of peptide-vectors that target the rodent and human LDLR. Initial screening of complex peptide libraries followed by chemical optimization led to the development of a family of short cyclic peptides (eight natural or non-natural residues) with distinct properties in terms of affinity, stability and biodistribution.

Real time two-photon microscopy experiments on mice demonstrated the ability of a lead peptide-vector to transport a non-permeable agent such as RhoRedX across the BBB and the blood–spinal cord barrier. As a further proof of concept, following intravenous (iv) administration in mice, peptide-vectors efficiently transported to the brain molecules such as opiate peptides or neuropeptides, all known to poorly cross the BBB. In particular, a vector-neurotensin conjugate is under preclinical development for its potential to induce pharmacological hypothermia with neuroprotective effects in acute excitotoxic neurodegeneration.

Some tumors including glioblastoma are associated with high-level expression of receptors involved in cell metabolism such as the LDLR. Conjugating our peptide-vectors to ^68^Gallium-NODAGA or -DOTA allowed PET imaging of glioblastoma in mouse brain. Others have shown that nanoparticles or liposomes functionalized with one of our lead peptide-vectors (peptide-22) permeate the BBB and exhibit higher glioma distribution than non-functionalized nanoparticles or liposomes; functionalized nanoparticles or liposomes loaded with paclitaxel and doxorubicin respectively elicit significantly prolonged life span of glioma-bearing mice [27, 28].

The lead peptide vectors also allowed uptake of vectorized protein cargos such as an antibody Fc fragment by brain endothelial cells and transport across an in vitro BBB model, and in vivo into the brain following iv administration in mice [29]. The peptide-vectors are currently assessed with industrial partners for their potential to transport therapeutic antibodies into the brain. In summary, we have developed a family of chemically optimized peptide-vectors that can be conjugated to a variety of different compounds such as small organic molecules, peptides, siRNAs and proteins including therapeutic antibodies. These peptide vectors bind the rodent and human LDLR and promote transport of cargos across the BBB and brain uptake in rodents. Such peptide-vectors appear promising for CNS delivery of different classes of drugs.

## Conclusions

From the variety of talks, it was clear that significant progress is being made in understanding control processes at brain barrier interfaces and in novel drug delivery methods. Modification of the normal barrier processes occurs in inflammatory situations through signalling cascades which can be manipulated, barrier gene expression can be modified with micro RNAs and control can be exerted over barrier transporter functions. Techniques for enhancing delivery of therapeutics to the CNS involve cleverly-devised delivery vehicles such as drug-carrying liposomes, endothelial-specific viral vectors, peptide conjugates, engineered protein capsules, nanoparticles, bi-specific antibodies and human monoclonal antibodies. Methods to test the efficacy of therapeutic delivery were discussed using both in vitro and in vivo techniques. Overall this was a stimulating conference bringing together scientists from a number of different disciplines.
