# Correction to: Current research into brain barriers and the delivery of therapeutics for neurological diseases: a report on CNS barrier congress London, UK, 2017

**DOI:** 10.1186/s12987-017-0086-x

**Published:** 2018-01-12

**Authors:** John Greenwood, Margareta Hammarlund-Udenaes, Hazel C. Jones, Alan W. Stitt, Roosmarijn E. Vandenbroucke, Ignacio A. Romero, Matthew Campbell, Gert Fricker, Birger Brodin, Heiko Manninga, Pieter J. Gaillard, Markus Schwaninger, Carl Webster, Krzysztof B. Wicher, Michel Khrestchatisky

**Affiliations:** 1 Institute of Ophthalmology, University College London, London, EC1V 9EL UK; 2 Department of Pharmaceutical Biosciences, Uppsala University, 751 24 Uppsala, Sweden; 3 Gagle Brook House, Chesterton, Bicester, OX26 1UF UK; 4 Centre for Experimental Medicine, Queen’s University Belfast, Belfast, Northern Ireland UK; 5 Department of Biomedical Molecular Biology, Ghent University, Ghent, Belgium; 6 VIB-UGent Center for Inflammation Research, VIB, Ghent, Belgium; 7 School of Life, Health and Chemical Sciences, Open University, Milton Keynes, UK; 8 Smurfit Institute of Genetics, Lincoln Place Gate, Trinity College Dublin, Dublin 2, Ireland; 9 Institute of Pharmacy and Molecular Biotechnology, Ruprecht-Karls University, Heidelberg, Germany; 10 Department of Pharmacy, Faculty of Health and Medical Sciences, University of Copenhagen, Copenhagen, Denmark; 11 NEUWAY Pharma GmbH, Ludwig-Erhard-Allee 2, 53175 Bonn, Germany; 12 2-BBB Medicines BV, Leiden, Netherlands; 13 Institute of Experimental and Clinical Pharmacology and Toxicology, University of Lübeck, Lübeck, Germany; 14 Antibody Discovery and Protein Engineering, MedImmune, Cambridge, UK; 15 Ossianix Inc., Stevenage, UK; 16 CNRS, NICN, Aix Marseille Univ, Marseille, France; 17 Vect-Horus, Faculte de Medecine Nord, 51 Boulevard Pierre Dramard, Marseille, France

## Correction to: Fluids Barriers CNS (2017) 14:31 10.1186/s12987-017-0079-9

After publication of the article [1], it has been brought to our attention that there are some errors in the formatting of names in the final version of the article.

Firstly, the article’s fifth author had their name spelt incorrectly. Previously included as “Roosmarijn E. Vandenbrouke”, the correct spelling is “Roosmarijn E. Vandenbroucke”. This error was present in the author list and page 3 of the article.

Furthermore, author names in the article references had formatting issues. This occurred in cases where “van” is part of the naming structure. In these cases the rest of the last name was included incorrectly as an initial. The references affected are listed below with their corrected author list.

- Reference #7 should read: Vandenbroucke RE, Dejonckheere E, Van Lint P, Demeestere D, Van Wonterghem E, Vanlaere I, et al.
- Reference #8 should read: Brkic M, Balusu S, Van Wonterghem E, Gorlé N, Benilova I, Kremer A, et al.
- Reference #9 should read: Balusu S, Van Wonterghem E, De Rycke R, Raemdonck K, Stremersch S, Gevaert K, et al.
- Reference #10 should read: Reijerkerk A, Lopez-Ramirez MA, van Het Hof B, Drexhage JA, Kamphuis WW, Kooij G, et al.
- Reference #22 should read: Loryan I, Sinha V, Mackie C, Van Peer A, Drinkenburg W, Vermeulen A, et al.
